# Comparative Analysis of Robotic and Laparoscopic Partial Nephrectomy: Insights Into Precision and Efficiency

**DOI:** 10.1155/aiu/5540611

**Published:** 2026-03-16

**Authors:** Murad Asali, Galeb Asali

**Affiliations:** ^1^ Urology Department, Barzilai Medical Center, Ben Gurion University of the Negev, Beer Sheva, Israel, bgu.ac.il; ^2^ Assuta Medical Center, Ben Gurion University of the Negev, Ramat HaHayal, Beer Sheva, Israel, bgu.ac.il; ^3^ Faculty of Medicine in Safed, Bar-Ilan University, Ramat Gan, Israel, biu.ac.il

**Keywords:** analysis partial nephrectomy, laparoscopic partial nephrectomy, occlusion the artery, occlusion the vein, robot versus laparoscopy, robotic partial nephrectomy

## Abstract

**Background:**

Partial nephrectomy represents a nephron‐sparing strategy aimed at preserving renal function while balancing increased technical complexity and potential oncological considerations. Innovations in minimally invasive surgery, especially laparoscopic (LPN) and robotic‐assisted partial nephrectomy (RPN), have markedly enhanced patient recovery durations and facilitated increased surgical precision. This study compares the outcomes of these approaches across key surgical parameters.

**Methods:**

This retrospective analysis included 205 patients who underwent LPN (143) or RPN (62) between 2008 and 2024. The parameters examined encompassed ischemia time, blood loss, tumor diameter (as assessed by CT and pathology), and postoperative recovery. Statistical methods: *t*‐tests, ANOVA, and Fisher’s exact tests, were utilized to assess group differences.

**Results:**

No significant differences were found in ischemia time (LPN: 22.3 min vs. RPN: 20.6 min, *p* = 0.391) or blood loss (LPN: 78.97 mL vs. RPN: 90.11 mL, *p* = 0.676). Tumor diameters were similar between groups for CT and pathology (*p* > 0.05). Analysis of bulldog usage revealed that both vein (*p* = 0.023) and artery (*p* = 0.046) clamps significantly reduced bleeding. However, bulldog utilization showed no significant impact on operating time (*p* > 0.05).

**Conclusion:**

This study demonstrates that, within a single‐surgeon experience, transition from LPN to RPN can be achieved safely, with comparable perioperative outcomes. These findings support feasibility rather than superiority of robotic surgery, and broader conclusions regarding patient benefit or cost require prospective randomized evaluation.

## 1. Introduction

Partial nephrectomy is a​ well‐known surgical treatment that preserves renal function while efficiently treating localized kidney malignancies [1].

In recent decades, surgical methods for partial nephrectomy have evolved substantially, from open procedures to minimally invasive techniques such as laparoscopic and robotic‐assisted approaches that prioritize patient recovery and precision [2]. These innovations have altered patient treatment by shortening recovery periods, decreasing complication rates, and increasing precision [3].

Laparoscopic partial nephrectomy (LPN), established decades ago, is recognized for its satisfactory outcomes [4]. However, it requires considerable technical expertise due to its reliance on rigid instruments and two‐dimensional imaging [5]. In contrast, robotic partial nephrectomy (RPN) incorporates state‐of‐the‐art technology, including three‐dimensional visualization and highly articulated instruments, which can enhance surgeon dexterity and accuracy, particularly in complex cases [6, 7].

While the technological capabilities of robotic systems are evident, it remains to be seen whether these advancements translate to better clinical outcomes than laparoscopic procedures. Furthermore, the greater costs of robotic surgery require careful evaluation of its practicality, especially in resource‐limited settings [7, 8].

This study compares LPN and RPN by assessing crucial surgical parameters such as ischemia time, blood loss, tumor diameters, and postoperative recovery, hoping to clarify the relative virtues and limits of these two techniques using statistical analysis and surgeon expertise, ultimately guiding evidence‐based decision‐making in nephron‐sparing surgery [9, 10].

While randomized controlled trials represent the optimal methodology for comparing surgical approaches, such data remain limited, and the present study should be interpreted as an observational analysis reflecting real‐world surgical transition by an experienced laparoscopic surgeon.

## 2. Methods

A retrospective analysis was performed on patients undergoing LPN and RPN for suspected kidney tumors by a single surgeon (M.A.). The study ran from 2008 to 2024 and collected data on patient demographics and surgical factors including ischemia time, blood loss, operating time, and tumor diameter measured by CT before the surgery and by pathology post partial nephrectomy. To compare the two groups, *t*‐tests, ANOVA, and Fisher’s exact tests were used. The study included 143 patients for laparoscopic and 62 for robotic procedures.

## 3. Results

Table 1 presents no significant differences in age, BMI, laterality, and mean renal score between groups (*p* > 0.05), with the exception of gender distribution, which varies significantly (*p* < 0.05).

**TABLE 1:** Characteristics.

Variable	Laparoscopic	Robotic	*p* value	Test
Age (years) (mean ± SD)	59.9 ± 10.5	62.0 ± 9.8	0.236	Fisher exact test
BMI (mean ± SD)	28.95 ± 4.81	28.82 ± 3.96	0.902	*T*‐test
Gender (M/F)	75/68	43/19	0.031	Fisher exact test
Laterality (R/L)	71/72	26/36	0.447	*T*‐test
Mean renal score	7.5 ± 1.2	7.8 ± 1.3	0.21	*T*‐test

Table 2 shows no significant differences in ischemia time, blood loss (mL), tumor diameter by CT (mm) and tumor diameter by pathology (mm), and hospital stay.

**TABLE 2:** Comparison of intra‐ and postoperative variables.

Variable	Laparoscopic (mean ± SD)	Robotic (mean ± SD)	*p* value
Ischemia time (min)	22.28 ± 13.82	20.57 ± 12.30	0.343
Blood loss (mL)	78.97 ± 120.78	90.11 ± 99.64	0.676
Operating time (min)	147.4 ± 29.44	160.62 ± 30.34	0.073
Tumor diameter by CT (mm)	26.54 ± 12.15	27.72 ± 14.71	0.581
Tumor diameter by pathology (mm)	24.58 ± 12.49	23.26 ± 11.61	0.420
Hospital stay (days)	3.3 ± 1.1	3.1 ± 0.9	0.339

## 4. Discussion

The statistical analysis in Tables 1 and 2 indicates no significant differences between the laparoscopic and robotic groups regarding age, BMI, blood loss, or duration of hospital stay. Some of these results correspond with earlier research studies, including Bhayani et al. and Choi et al., which reported similar perioperative outcomes for minimally invasive nephrectomy methods but shorter hospital stay [1, 2].

Nonetheless, in this study, robotic procedures generally exhibit somewhat extended operating durations, perhaps indicative of the time necessary for robotic configuration and advanced maneuvers, contrary to the results published by Kalifeh et al. [3]. The surgeon’s (M.A.) extensive experience in laparoscopic surgery likely influenced the comparable outcomes between the two procedures, since surgeon expertise is a critical determinant of optimal results [5].

Table 3 reveals significant disparities in tumor diameter measured by CT and that documented in pathology across both groups (*p* < 0.05). This suggests that imaging amplifies tumor size compared to pathological assessment, which is why surgeons endeavor to create techniques for more precise resection utilizing a 3D model [11].

**TABLE 3:** Comparison of tumor diameter by CT and pathology.

Surgical technique	Measurement type	Mean (mm)	Std. dev. (mm)	*p* value
Laparoscopic	Diameter by CT	26.54	12.15	
Laparoscopic	Diameter‐pathology	24.58	12.49	< 0.001

Robotic	Diameter by CT	27.72	14.71	
Robotic	Diameter‐pathology	23.26	11.61	0.0014

Robotic procedures exhibited marginally more variability in CT measurements (SD = 14.71), possibly attributable to a higher proportion of complex cases. These disparities may also indicate the significance of surgical precision, as laparoscopic proficiency can reduce variability and guarantee consistent results [12].

Table 4 presents the ANOVA results (*F* = 2.72, *p* = 0.044), indicating significant differences among the four groups (laparoscopic‐CT/laparoscopic‐pathology and robotic‐CT/robotic‐pathology). These disparities underscore the intrinsic discrepancies in imaging and pathology assessments.

**TABLE 4:** ANOVA test results for tumor diameter by CT and pathology.

Groups compared	*F*‐statistic	*p* value
Laparoscopic‐CT, laparoscopic‐pathology, robotic‐CT, robotic‐pathology	2.72	0.044

The surgeon’s expertise in laparoscopic procedures may have reduced disparities between imaging and pathology, highlighting the essential role of surgical ability in obtaining dependable outcomes.

Bulldog usage in veins and arteries (Table 5) was significant. Vein bulldog had a statistically significant effect on bleeding (*p* = 0.023), suggesting it may affect surgical hemostatic control. Hemorrhaging was also linked to artery bulldog use (*p* = 0.046).

**TABLE 5:** Comparison of complications and evaluation of the use versus nonuse of bulldog clamps on the renal artery or renal vein.

Parameter	Test type	*p* value
Complications	Fisher exact test	0.099
Vein bulldog	Fisher exact test	0.023
Artery bulldog	Fisher exact test	0.046

The study found no significant influence of bulldog use on operating time (*p* > 0.05), despite bleeding differences. Clamps effectively regulate hemorrhage without prolonging the process. These findings support past data showing bulldog clamps can provide hemostasis without prolonging the surgery [13].

The disparities in the utilization of vein and artery bulldogs (Table 5) indicate a greater propensity for robotic procedures to employ these instruments (*p* < 0.05), highlighting the precision and control benefits of robotic surgery. The absence of notable differences in bleeding or blood transfusion rates (*p* > 0.05) indicates that both procedures offer sufficient hemostatic control when performed by skilled surgeons. The surgeon’s (M.A.) proficiency in advanced laparoscopic techniques may explain the comparable bleeding outcomes, as skill might counterbalance the technical benefits conferred by robotic systems.

## 5. General Considerations

Although laparoscopic and robotic procedures yield comparable clinical results, robotic systems may provide unique benefits, including improved dexterity and visualization, making them a favored option in complex cases. In intricate scenarios, robotic systems excel due to their unparalleled precision and control. The 3D visualization and articulating devices facilitate more precise dissection and suturing, as evidenced by research conducted by Leow et al. [9].

Furthermore, the ergonomic design of robotic platforms alleviates physical strain on surgeons, enhancing concentration and endurance during prolonged surgical procedures [14].

A significant benefit is the ability to normalize outcomes across different degrees of surgical proficiency. Robotic systems alleviate the steep learning curve linked to laparoscopic methods, enabling less experienced surgeons to attain equivalent outcomes [15, 16]. Although these studies [15, 16] have suggested that robotic platforms may mitigate learning curve effects, the present single‐surgeon retrospective analysis does not allow definitive conclusions regarding learning curve advantages, and our observations should therefore be interpreted as descriptive rather than inferential in this regard.

Moreover, robotic surgery provides enhanced dexterity for tumor access in anatomically complex locations, as evidenced by research conducted by Hinata et al. and Buffi et al. [7, 10].

Ultimately, robotic surgery represents the future of minimally invasive techniques. The proliferation of these technologies necessitates investment in robotic platforms to ensure adaptation to advancing surgical techniques and patient requirements. This progressive viewpoint corresponds with the conclusions of Rogers et al., who highlighted the necessity of incorporating modern technologies into surgical practice [17].

Both methodologies exhibit significant advantages, with robotic surgery offering ergonomic benefits and enhanced dexterity, whereas laparoscopic techniques reveal cost‐effectiveness and comparable clinical results when performed by skilled surgeons. Khalifeh et al. observed that the selection of technique frequently relies on institutional resources and the complexity of the case [3].

## 6. Limitations

The limited sample size for robotic procedures may compromise the statistical power to identify minor variations.

### 6.1. Evaluating Results: Laparoscopic Versus Robotic Techniques

The comparison of LPN with RPN reveals that both methods yield similar clinical results in critical metrics, including ischemia duration, blood loss, and tumor size. Nevertheless, subtle distinctions exist that illustrate the intrinsic strengths and weaknesses of each method.

1. Ischemia time: The mean ischemia time was marginally reduced in the robotic group (20.6 min) relative to the laparoscopic group (22.3 min), while the difference lacked statistical significance (*p* = 0.391). This finding corroborates the research by Leow et al., indicating that the superior precision of robotic systems may enable expedited vascular control, especially in complex cases [9]. Nonetheless, the absence of relevance may also suggest that proficient laparoscopic surgeons can attain similar durations, as evidenced by studies like Khalifeh et al. [3].
2. Blood loss: Both groups had negligible differences in average blood loss (LPN: 78.97 mL vs. RPN: 90.11 mL, *p* = 0.676). This outcome demonstrates the hemostatic effectiveness of both methods when performed by experienced surgeons. Studies such as Choi et al. and Khalifeh et al. highlight that surgeon proficiency substantially reduces intraoperative hemorrhage regardless of the surgical technique employed [2, 3].
3. Tumor diameter: Imaging and pathological assessments demonstrated no significant changes between the groups (CT: *p* = 0.581; pathology: *p* = 0.420). This finding indicates that both methods can proficiently handle tumors of comparable size and complexity. The variability in CT measures in the robotic group (SD = 14.71) may suggest a greater prevalence of anatomically complex malignancies.

### 6.2. Advantages of Robotic Surgery

RPN provides significant advantages, especially for contemporary surgical requirements. Robotic technologies provide increased three‐dimensional imaging and improved dexterity, hence enhancing surgical precision. This feature is particularly beneficial for targeting tumors in difficult anatomical locations, reducing inadvertent harm to adjacent organs [4, 7].

The ergonomic design of robotic systems diminishes surgeon fatigue, facilitating the execution of prolonged and complex procedures with greater focus and accuracy [14].

Another significant advantage is the robotic system’s scalability. The intuitive nature of robotic controls allows novice surgeons to obtain results comparable to expert laparoscopic surgeons. This accessibility helps to standardize care and increase the availability of minimally invasive procedures, especially in institutions with limited laparoscopic experience [18].

**FIGURE 1:**
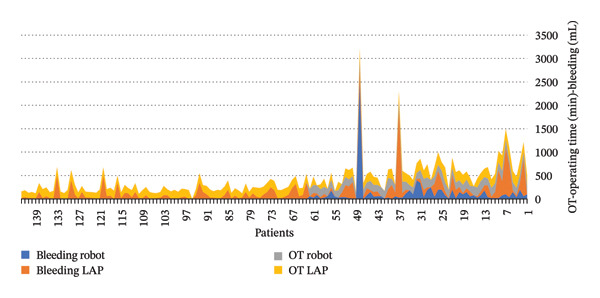
Bleeding and duration in laparoscopic versus robotic surgery.

As shown in Figure 1, four variables are analyzed: bleeding in laparoscopic versus robotic surgery and duration of laparoscopic versus robotic surgeries over time. This figure reveals the following:

1. Trends in duration:
  • Laparoscopic surgery duration decreases over time, indicating an improvement in technique with experience.
  • Robotic surgery duration remains relatively stable, reflecting a less steep learning curve.
2. Trends in bleeding:
  • A reduction in bleeding is observed in laparoscopic surgeries over time.
  • Robotic surgeries maintain consistent low bleeding levels, highlighting the precision of robotic systems.

These trends suggest that

- Laparoscopic procedures have a learning curve that can be improved with experience.
- Robotic surgeries have a stable learning curve with predictable outcomes.
- The data may be influenced by a selection bias, as robotic surgeries were most likely introduced after years of laparoscopic experience.

These observations underline the advantages of robotic systems in standardizing outcomes while emphasizing the role of experience in optimizing laparoscopic procedures [16, 18].

### 6.3. Advantages of Laparoscopic Surgery

LPN, on the other hand, continues to be relevant due to its low cost and widespread availability:

• Cost efficiency: LPN is a cost‐effective alternative to robotic platforms, making it ideal for resource‐constrained environments [8].
• Established efficacy: Decades of evidence support LPN as a safe and effective treatment for kidney malignancies [3, 19].

## 7. Limitations and Future Directions

The study’s limitations include a smaller sample size for robotic cases and potential biases related to surgeon expertise. Future research should incorporate multi‐institutional data and randomized trials to validate these findings and explore long‐term renal functional outcomes.

## 8. Conclusion

This study demonstrates that, within a single‐surgeon experience, transition from LPN to RPN can be achieved safely, with comparable perioperative outcomes. These findings support feasibility rather than superiority of robotic surgery, and broader conclusions regarding patient benefit or cost require prospective randomized evaluation.

## Author Contributions

M.A.: conceptualization, visualization, investigation, supervision, original draft preparation, data curation, and methodology.

G.A.: data, English editing, and original draft preparation.

## Funding

No funding was received for this study.

## Disclosure

All authors have read and approved the final version of the manuscript.

## Ethics Statement

All patients provided written informed consent prior to the procedure.

Helsinki No. 0087‐24‐ASMC.

Date of discussion and approval by the Helsinki Committee: 10.7.2025 and 10.8.2025.

## Consent

All patients provided written informed consent prior to the procedure.

## Conflicts of Interest

The authors declare no conflicts of interest.

## Data Availability

All the data saved will be provided on request. The data that support the findings of this study are not publicly available due to patients being in private hospitals but are available from the corresponding author—Dr. M.A.

## References

[1] Bhayani S. B. and Das N. , Robotic Assisted Laparoscopic Partial Nephrectomy for Suspected Renal Cell Carcinoma: Retrospective Review of Surgical Outcomes of 35 Cases, BMC Surgery. (2008) 8, no. 1, 10.1186/1471-2482-8-16, 2-s2.0-53649084942. PMC2564900 18816400

[2] Choi J. E. , You J. H. , Kim D. K. , Rha K. H. , and Lee S. H. , Comparison of Perioperative Outcomes Between Robotic and Laparoscopic Partial Nephrectomy: A Systematic Review and Meta-Analysis, European Urology. (2015) 67, no. 5, 891–901, 10.1016/j.eururo.2014.12.028, 2-s2.0-84926205878. 25572825

[3] Khalifeh A. , Autorino R. , Hillyer S. P. et al., Comparative Outcomes and Assessment of ‘Trifecta’ in 500 Robotic and Laparoscopic Partial Nephrectomies: A Single Surgeon Experience, The Journal of Urology. (2013) 189, no. 4, 1236–1242, 10.1016/j.juro.2012.10.021, 2-s2.0-84875955561. 23079376

[4] Abreu A. L. , Berger A. K. , Aron M. et al., Minimally Invasive Partial Nephrectomy for Single Versus Multiple Renal Tumors, Urology Times. (2013) 189, no. 2, 462–467, 10.1016/j.juro.2012.09.039, 2-s2.0-84872165992. 23253959

[5] Porpiglia F. , Volpe A. , Billia M. , and Scarpa R. M. , Laparoscopic Versus Open Partial Nephrectomy: Analysis of the Current Literature, European Urology. (2008) 53, no. 4, 732–742, 10.1016/j.eururo.2008.01.025, 2-s2.0-39549092419. 18222599

[6] Wang Y. , Teng Q. , Dai Z. et al., Three-Dimensional Visualization Techniques Improve Surgical Decision Making of Robotic-Assisted Partial Nephrectomy, eCollection. (2024) 10, no. 21, 10.1016/j.heliyon.2024.e38806. PMC11550665 39524740

[7] Hinata N. , Shiroki R. , Tanabe K. et al., Robot-Assisted Partial Nephrectomy Versus Standard Laparoscopic Partial Nephrectomy for Renal Hilar Tumor: A Prospective Multi-Institutional Study, International Journal of Urology. (2021) 28, no. 4, 382–389, 10.1111/iju.14469. 33368639

[8] Alemozaffar M. , Chang S. L. , Kacker R. , Sun M. , DeWolf W. C. , and Wagner A. A. , Comparing Costs of Robotic, Laparoscopic, and Open Partial Nephrectomy, Journal of Endourology. (2013) 27, no. 5, 560–565, 10.1089/end.2012.0462, 2-s2.0-84885925194. 23130756

[9] Leow J. J. , Heah N. H. , Chang S. L. , Chong Y. L. , and Png K. S. , Outcomes of Robotic Versus Laparoscopic Partial Nephrectomy: An Updated Meta-Analysis of 4,919 Patients, The Journal of Urology. (2016) 196, no. 5, 1371–1377, 10.1016/j.juro.2016.06.011, 2-s2.0-84992395871. 27291654

[10] Buffi N. M. , Saita A. , Lughezzani G. et al., Robot-Assisted Partial Nephrectomy for Complex (PADUA Score ≥ 10) Tumors: Techniques and Results From a Multicenter Experience at Four High-Volume Centers, European Urology. (2020) 77, no. 1, 95–100, 10.1016/j.eururo.2019.03.006, 2-s2.0-85062996241. 30898407

[11] Xu L. , Li X. , Zhang Y. et al., A Novel Preoperative Evaluation Technique for Partial Nephrectomy: Three-Dimensional Extended Renal Tumor Plane, World Journal of Urology. (2024) 43, no. 1, 10.1007/s00345-024-05395-2. 39714534

[12] Cei F. , Larcher A. , Rosiello G. et al., Preoperative Risk Calculator for the Probability of Completing Nephron Sparing for Kidney Cancer, Urologic Oncology. (2024) 42, no. 8, 247.e21–247.e27, 10.1016/j.urolonc.2024.01.029. 38644109

[13] Sukumar S. , Petros F. , Mander N. , Chen R. , Menon M. , and Rogers C. G. , Robotic Partial Nephrectomy Using Robotic Bulldog Clamps, Journal of the Society of Laparoendoscopic Surgeons. (2011) 15, no. 4, 520–526, 10.4293/108680811X13176785204274, 2-s2.0-84861474389. 22643509 PMC3340963

[14] Farr D. , Can Robotic Surgery Contribute to Surgeon Wellness?, The American Journal of Surgery. (2024) 237, 10.1016/j.amjsurg.2024.115831. 39013703

[15] Livinti I. , Osofsky R. , Burgamy A. , Loehrke-Sichhart L. , Mikhail M. , and Shah A. , Laparoscopic Versus Robotic Surgery Learning Curves, Journal of Minimally Invasive Gynecology. (2015) 22, no. 6, S8–S9, 10.1016/j.jmig.2015.08.031. 27679343

[16] Larcher A. , Muttin F. , Peyronnet B. et al., The Learning Curve for Robot-Assisted Partial Nephrectomy: Impact of Surgical Experience on Perioperative Outcomes, European Urology. (2019) 75, no. 2, 253–256, 10.1016/j.eururo.2018.08.042, 2-s2.0-85053671696. 30243798

[17] Rogers C. G. , Sukumar S. , Patel M. N. et al., Robotic Partial Nephrectomy: A Multi-Institutional Analysis, Journal of Robotic Surgery. (2008) 2, no. 3, 141–143, 10.1007/s11701-008-0098-2, 2-s2.0-51549107078. 27628250

[18] Panaiyadiyan S. and Kumar R. , Outcomes of Robotic Surgery for Low-Volume Surgeons, Société Internationale d’Urologie Journal. (2022) 3, no. 5, 323–330, 10.48083/PPSC8658.

[19] Bravi C. A. , Dell’Oglio P. , Pecoraro A. et al., Surgical Experience and Functional Outcomes After Laparoscopic and Robot-Assisted Partial Nephrectomy: Results From a Multi-Institutional Collaboration, Journal of Clinical Medicine. (2024) 13, no. 19, 10.3390/jcm13196016. PMC11477761 39408076

